# Plasma Heme Oxygenase-1 Concentration Is Elevated in Individuals with Type 2 Diabetes Mellitus

**DOI:** 10.1371/journal.pone.0012371

**Published:** 2010-08-25

**Authors:** Wei Bao, Fangfang Song, Xiangyang Li, Shuang Rong, Wei Yang, Muxun Zhang, Ping Yao, Liping Hao, Nianhong Yang, Frank B. Hu, Liegang Liu

**Affiliations:** 1 Department of Nutrition and Food Hygiene and MOE Key Lab of Environment and Health, School of Public Health, Tongji Medical College, Huazhong University of Science and Technology, Wuhan, People's Republic of China; 2 Department of Epidemiology and Biostatistics, Tianjin Medical University Cancer Institute and Hospital, Tianjin, People's Republic of China; 3 Department of Internal Medicine, Institute of Endocrinology, Tongji Hospital, Tongji Medical College, Huazhong University of Science and Technology, Wuhan, People's Republic of China; 4 Departments of Nutrition and Epidemiology, Harvard School of Public Health, Boston, Massachusetts, United States of America; 5 Channing Laboratory, Department of Medicine, Brigham and Women's Hospital, and Harvard Medical School, Boston, Massachusetts, United States of America; National Institute of Child Health and Human Development/National Institutes of Health, United States of America

## Abstract

**Background:**

Circulating concentrations of heme oxygenase-1 (HO-1) have been recently reported to be elevated in several chronic disorders. However, no study has ever examined the association between circulating HO-1 concentrations and type 2 diabetes mellitus (T2DM).

**Methods and Findings:**

581 cases with newly-diagnosed T2DM (New-T2DM) and 611 comparison controls were recruited in this two-phase case-control study, comprising 420 cases and 429 controls collected in the first phase study and 161 cases and 182 controls in the second phase replication study. Analyses, using both separated data and combined data from the two-phase studies, show that plasma HO-1 concentrations were significantly increased in New-T2DM cases compared to controls (*P*<0.001). Plasma HO-1 concentrations were significantly correlated with plasma glucose concentrations, HOMA-beta and HOMA-IR (*P*<0.001). After adjustment for age, sex, BMI and family history of diabetes, the ORs for New-T2DM in the highest quartile of plasma HO-1 concentrations, compared with the lowest, was 8.23 (95% CI 5.55–12.21; *P* for trend <0.001). The trend remained significant after additional adjustment for fasting plasma glucose/insulin, HOMA-beta/HOMA-IR, TC/TG, smoking, drinking and history of hypertension, and even in further stratification analysis by age, sex, BMI, smoking, drinking and history of hypertension.

**Conclusions:**

Elevated plasma HO-1 concentrations are associated with higher ORs for New-T2DM, which add more knowledge regarding the important role of oxidative stress in T2DM. More consequent studies were warranted to confirm the clinical utility of plasma HO-1, especially in diagnosis and prognosis of T2DM and its complications.

## Introduction

Although the underlying mechanisms for the pathogenesis of type 2 diabetes mellitus (T2DM) still remain to be fully determined, growing evidence has suggested that oxidative stress, induced by hyperglycemia, glucose fluctuations [1] and possibly by free fatty acid (FFA) [2], contributes to the development of T2DM and diabetic complications [3]–[5]. Among a panel of potential candidate genes related to oxidative stress, heme oxygenase-1 (HO-1) has drawn much attention as an “emerging molecule” with potent antioxidant, anti-inflammatory, and anti-proliferative effects [6].

HO-1, also known as heat shock protein 32 (Hsp32), is the inducible isoform of heme oxygenase that catalyzes the NADPH-dependent decomposition of heme to carbon monoxide (CO), ferrous iron, and biliverdin [7]. HO-1 expression is highly responsive to a broad spectrum of chemical and physical stress agents, such as hydrogen peroxide, heavy metals, UVA irradiation, hypoxia, hyperoxia, pro-inflammatory cytokines and heme itself, thus it has also been regarded as a biomarker for cell stress status [8]. Given the potential physiological importance of HO-1 in mediating cellular homeostasis, its role in T2DM has been investigated in considerable research. It is interesting that although high glucose exposure failed to induce the classical antioxidant enzymes, such as superoxide dismutase, catalase and glutathione peroxidase, in pancreatic beta cells [9], it resulted in a remarkable rise in both HO-1 gene expression and enzyme activities in the islets, in parallel with hyperglycemia-induced intracellular peroxide levels [10], [11]. Another line of research examined the effects of the duration of hyperglycemia on HO-1 gene expression in islets of partially pancreatectomized rats. In rats with higher levels of hyperglycemia (>150 mg/dl), blood glucose concentrations continued to increase while HO-1 expressions in islets were decreased over time [12], which is similar with the results in our previous study in alloxan-induced diabetic mice [13]. These studies suggest that HO-1 induction may be an early-phase event responding to high glucose challenge in T2DM.

Recently, elevated serum or plasma HO-1 concentrations have been observed in several chronic disorders [14]–[16]. In the other hand, HO-1 expressions in several types of cells or tissues of individuals with T2DM have been previously reported by our group and others [17]–[21]. However, no study has examined whether circulating HO-1 concentrations are related to T2DM. Therefore, we conducted this case-control study to investigate the association between plasma HO-1 concentrations and T2DM in newly diagnosed cases (New-T2DM), and moreover to quantify this association while taking into account the effect of important confounding factors and in the context of age, sex and body mass index (BMI) of the study subjects.

## Methods

### Study population

The study protocol was approved by the Medical Ethics Committee of Tongji Medical College and performed according to the declaration of Helsinki. Written informed consent was obtained from all individuals. A two-phase case-control design, including an original study and an independent replication study, were applied in the current study to obtain a total of 581 cases with New-T2DM and 611 comparison controls in two independent operations.

In the first phase, we performed an original case-control study to include 420 cases with New-T2DM and 429 controls. All cases were recruited from patients who, for the first time, received a diagnosis of T2DM at the outpatient clinics of Department of Endocrinology of Tongji Hospital affiliated to Tongji Medical College during the period of December 2004 to December 2007. Comparison controls were drawn from an unselected group of population that underwent for a routine health examination in the same hospital. The enrolled controls have a similar sex and age distribution as to the cases. All the diabetes cases met the respective diagnostic criteria recommended by World Health Organization in 1999 incorporating both fasting plasma glucose (FPG) and a 2-h oral glucose tolerance test (OGTT; 75 g of glucose) [22]. We included only individuals with New-T2DM as cases to avoid possible confounding effects by anti-diabetic medications. Moreover, most New-T2DM cases were at an earlier stage of T2DM progression. For both the cases and controls, we restricted the study subjects to only individuals who were aged ≥30 years, BMI <40 kg/m^2^, no early history of diagnosed diabetes, nor any other clinically systemic diseases, acute or chronic inflammatory diseases, acute respiratory infection, and cancer.

In the second phase of the present study, we recruited another 343 participants, including 161 cases and 182 controls, with a purpose of replication of our first phase study. The replication study was carried out about two months after we finished the original study. These two studies shared the same diagnostic standard and the same inclusion/exclusion criteria.

A standard questionnaire was used to collect information about age, sex, smoking, alcohol consumption, hypertension, and family history of diabetes in their first-degree relatives. Anthropometric measurements included height (m), weight (kg), and blood pressure (mmHg) using standardized techniques. Body mass index (BMI) was calculated as weight (kg)/square of height (m^2^). All subjects underwent a complete physical examination in the morning after an overnight fast; venous blood samples were drawn from an antecubital vein into heparinized tubes for plasma separation.

### Laboratory Measurements

Plasma levels for biochemical parameters, including fasting plasma glucose (FPG), 2-h post-glucose load (OGTT2h), fasting plasma insulin (FPI), total cholesterol (TC) and triglycerides (TG), were measured as previously described [23]. Intra- and inter-assay coefficients of variation were <4% for all these assays.

Homeostasis model assessment of beta cell function (HOMA-beta) and insulin resistance (HOMA-IR) were employed to assess the status of insulin secretion and insulin action, respectively. HOMA-beta  = 20×FPI (µU/ml)/[FPG (mmol/L)−3.5], HOMA-IR  =  FPG (mmol/L) ×FPI (µU/ml)/22.5 [24].

Plasma HO-1 concentrations were determined by enzyme-linked immunosorbent assay [14]–[16] using commercially available sandwich kits (EKS-800, Stressgen Bioreagents; now Assay Designs, Ann Arbor, MI, USA). HO-1 concentrations from the samples were quantitated by interpolatig absorbance readings at 450 nm in a microplate reader (Synergy 2 multimode microplate reader, Bio-Tek Instrument, Winooski, VT, USA) from a standard curve generated with the calibrated HO-1 protein standard provided. The intra-assay and inter-assay coefficient of variation of the HO-1 ELISA kit has been determined to be <10%.

### Statistical Analysis

Statistical analyses were performed using SPSS for windows software version 12.0 (SPSS Inc, Chicago, IL, USA). Descriptive statistics were calculated for all demographic and clinical characteristics of the study subjects. Comparisons between diabetes cases and controls were performed by Chi-square (categorical variables), t test (continuous variables, normal distribution) or Mann-Whitney U test (continuous variables, skewed distribution). Because plasma HO-1 concentrations showed a skewed distribution, Spearman correlation coefficients were used to describe the correlation between HO-1 and the continuous variables of interest. After adjustment for age, sex and body mass index (BMI), partial Spearman correlation coefficients were calculated between HO-1 and other variables.

Multivariate logistic regression analysis was used to evaluate the independent association of plasma HO-1 concentration with the likelihood of New-T2DM. Hosmer-Lemeshow goodness-of-fit tests were used to evaluate the appropriate model fit. Adjustments were made for age and sex (Model 1); additionally made for BMI and family history of diabetes (Model 2); additionally made for FPG and FPI (Model 3a) and HOMA-IR and HOMA-beta (3b). For calculation of the odds ratios (ORs) for New-T2DM, plasma HO-1 concentrations were categorized in quartiles according to the control group: category 1, <0.63 ng/ml; category 2, 0.63–1.14 ng/ml, category 3, 1.14–2.06 ng/ml and category 4, ≥2.06 ng/ml. Then we conducted stratified analyses by age (<55, ≥55 y), sex, and overweight (BMI<24, ≥24 kg/m^2^) [25]. Likelihood ratio test was used to examine statistical significance of interactions between the above variables and HO-1 concentrations.

We performed all above described analyses for the data collected in the two-phase studies, both separately and with the combined data. Due to the highly consistency on almost all study variables and observed correlations between HO-1 and other variables, we used combined data for the later multivariate statistical analysis. All reported *P* values are 2-sided and *P*<0.05 were considered to be statistically significant.

## Results

Demographic and clinical characteristics of the two-phase study subjects are shown separately in Table 1 (Original Study) and Table 2 (Replication Study), respectively, and then with the combined data in Table 3 (Combined Study). In both the original study and the replication study, the individuals with New-T2DM, compared to controls, had higher BMI, higher prevalence of family history of diabetes and hypertension, and higher levels of triglyceride, total cholesterol, FPG, OGTT 2h and plasma HO-1. When looking at the insulin sensitivity indexes, we, as expected, observed a lower HOMA-beta but a higher HOMA-IR in diabetes cases than that in the controls (Tables 1, 2 and 3). Plasma HO-1 concentrations were significantly increased in patients with New-T2DM compared with controls. In the original study, Median (interquartile range, IQR) is 2.42 (1.39–3.90) ng/ml vs. 1.11 (0.63–2.06) ng/ml in diabetes cases vs. controls, respectively (*P*<0.001); and in the replication study, that is 2.62 (1.81–3.97) vs. 1.22 (0.61–2.06), respectively (P<0.001) (Tables 1, 2 and 3).

**Table 1:** Characteristics of the Newly Diagnosed Type 2 Diabetes Cases and Controls in the Original Study.

Characteristics	Diabetes Cases	Controls	*P* Value
Number of Subjects	420	429	
Age, years	51.12 (10.74)	51.90 (11.82)	0.310
Male, n (%)	246 (58.57)	230 (53.61)	0.146
BMI, kg/m^2^	24.69 (22.90−26.74)	23.94 (21.97−25.85)	<0.001
Smoke, n (%)	203 (48.33)	172 (40.09)	0.016
Alcohol, n (%)	238 (56.67)	190 (44.29)	<0.001
Family History of Diabetes, n (%)	98 (23.33)	67 (15.62)	0.005
Hypertention, n (%)	172 (40.95)	137 (31.93)	0.006
TC (mmol/L)	5.29 (4.34−6.34)	4.66 (4.02−5.28)	<0.001
TG (mmol/L)	1.76 (1.16−2.63)	1.26 (0.86−1.81)	<0.001
FPG (mmol/L)	8.65 (7.30−11.33)	5.20 (4.70−5.84)	<0.001
FPI (µU/ml)	10.07 (6.36−14.89)	10.14 (7.77−12.40)	0.983
OGTT2h (mmol/L)	15.43 (13.35−17.34)	8.57 (7.86−9.60)	<0.001
HOMA-beta	37.17 (19.63−70.93)	119.55 (80.94−184.58)	<0.001
HOMA-IR	3.86 (2.55−5.97)	2.31 (1.78−3.06)	<0.001
HO-1 (ng/ml)	2.42 (1.39−3.90)	1.11 (0.63−2.06)	<0.001

Abbreviations: BMI, body mass index; TC, total cholesterol; TG, triglycerides; FPG, fasting plasma glucose; FPI, fasting plasma insulin; OGTT2h, 2-h post-glucose load; HOMA-beta, homeostasis model assessment of beta cell function; HOMA-IR, homeostasis model assessment of insulin resistance.

Data are presented as number (percentage) for categorical data, mean (standard deviation) for parametrically distributed data or median (interquartile range) for nonparametrically distributed data.

**Table 2:** Characteristics of the Newly Diagnosed Type 2 Diabetes Cases and Controls in the Replication Study.

Characteristics	Diabetes Cases	Controls	*P* Value
Number of Subjects	161	182	
Age, years	50.10 (8.17)	49.79 (9.23)	0.741
Male, n (%)	93 (57.76)	98 (53.85)	0.467
BMI, kg/m^2^	23.31 (21.94−24.89)	22.72 (20.80−24.89)	0.354
Smoke, n (%)	59 (36.65)	83 (45.60)	0.093
Alcohol, n (%)	71 (44.10)	77 (42.31)	0.738
Family History of Diabetes, n (%)	36 (22.36)	20 (10.99)	0.004
Hypertention, n (%)	47 (29.19)	47 (25.82)	0.485
TC (mmol/L)	4.03 (3.43−4.82)	4.33 (3.86−4.90)	0.003
TG (mmol/L)	1.35 (0.85−2.00)	1.22 (0.75−1.62)	0.059
FPG (mmol/L)	10.25 (8.32−12.86)	4.86 (4.30−5.60)	<0.001
FPI (µU/ml)	6.81 (4.50−10.61)	8.33 (6.17−12.23)	0.010
OGTT2h (mmol/L)	18.95 (16.53−22.54)	8.26 (7.73−8.82)	<0.001
HOMA-beta	20.70 (10.85−37.43)	87.02 (63.31−112.30)	<0.001
HOMA-IR	3.13 (2.07−4.76)	1.19 (0.66−2.04)	<0.001
HO-1 (ng/ml)	2.62 (1.81−3.97)	1.22 (0.61−2.06)	<0.001

Data are presented as number (percentage) for categorical data, mean (standard deviation) for parametrically distributed data or median (interquartile range) for nonparametrically distributed data.

**Table 3:** Characteristics of the Newly Diagnosed Type 2 Diabetes Cases and Controls in the Combined Population from both the Original Study and Replication Study.

Characteristics	Diabetes Cases	Controls	*P* Value
Number of Subjects	581	611	
Age, years	50.83 (10.10)	51.27 (11.14)	0.477
Male, n (%)	339 (58.35)	328 (53.68)	0.105
BMI, kg/m^2^	24.22 (22.49−26.17)	23.67 (21.67−25.54)	<0.001
Smoke, n (%)	262 (45.09)	255 (41.73)	0.242
Alcohol, n (%)	309 (53.18)	267 (43.70)	0.001
Family History of Diabetes, n (%)	134 (23.06)	87 (14.24)	<0.001
Hypertention, n (%)	219 (37.69)	184 (30.11)	0.006
TC (mmol/L)	4.93 (3.96−6.03)	4.56 (3.97−5.15)	<0.001
TG (mmol/L)	1.61 (1.05−2.46)	1.25 (0.83−1.78)	<0.001
FPG (mmol/L)	9.17 (7.47−11.83)	5.10 (4.60−5.79)	<0.001
FPI (µU/ml)	8.96 (5.86−13.40)	9.24 (6.18−11.98)	0.287
OGTT2h (mmol/L)	16.20 (13.71−18.50)	8.44 (7.83−9.45)	<0.001
HOMA-beta	32.52 (16.47−62.70)	106.63 (72.68−169.20)	<0.001
HOMA-IR	3.60 (2.36−5.48)	2.05 (1.36−2.87)	<0.001
HO-1 (ng/ml)	2.51 (1.53−3.93)	1.14 (0.62−2.06)	<0.001

Data are presented as number (percentage) for categorical data, mean (standard deviation) for parametrically distributed data or median (interquartile range) for nonparametrically distributed data.

Table 4 shows correlations between plasma HO-1 concentrations and relevant variables. Plasma HO-1 concentrations were significantly correlated with plasma glucose concentrations, HOMA-beta, and HOMA-IR. In the original study, Spearman correlation coefficient (r) is 0.351 for FPG, 0.317 OGTT2h, −0.285 for HOMA-beta, and 0.192 for HOMA-IR, respectively (all *P*<0.001); and in the replication study, r is 0.529 for FPG, 0.400 for OGTT2h, −0.316 for HOMA-beta, and 0.481 for HOMA-IR, respectively (all *P*<0.001). In partial correlation analysis, after adjustment for age, sex and BMI, these correlations between plasma HO-1 concentrations and other variables above were attenuated but they remained significant in both studies. Moreover, when we performed the above analyses using the combined data from the original study and replication study, the trend is still unchanged (Table 4).

**Table 4:** Spearman Correlation Coefficients between Plasma HO-1 Levels and Other Variables in the Study Subjects.

	Unadjusted		Adjusted for Age, Sex and BMI	
Variables	r	*P* value	r	*P* value
**Original Study**				
Age	−0.055	0.112	/	/
BMI	0.151	0.003	/	/
TC	0.085	0.013	0.028	0.420
TG	0.104	0.002	0.045	0.198
FPG	0.351	<0.001	0.226	<0.001
FPI	0.003	0.940	0.019	0.577
OGTT2h	0.317	<0.001	0.209	<0.001
HOMA-beta	−0.285	<0.001	−0.188	<0.001
HOMA-IR	0.192	<0.001	0.107	0.002
**Replication Study**				
Age	0.255	<0.001	/	/
BMI	0.044	0.415	/	/
TC	−0.076	0.161	−0.035	0.521
TG	0.024	0.657	0.017	0.755
FPG	0.529	<0.001	0.311	<0.001
FPI	0.307	<0.001	0.269	<0.001
OGTT2h	0.400	<0.001	0.286	<0.001
HOMA-beta	−0.316	<0.001	−0.153	0.005
HOMA-IR	0.481	<0.001	0.346	<0.001
**Combined Study**				
Age	0.026	0.362	/	/
BMI	0.112	<0.001	/	/
TC	0.033	0.260	0.011	0.708
TG	0.078	0.007	0.037	0.211
FPG	0.406	<0.001	0.258	<0.001
FPI	0.060	0.038	0.064	0.029
OGTT2h	0.349	<0.001	0.234	<0.001
HOMA-beta	−0.292	<0.001	−0.179	<0.001
HOMA-IR	0.255	<0.001	0.153	<0.001

The analyses are based on the data from both cases and controls.

As mentioned above, most of the study variables and observed correlations between HO-1 and other variables were highly consistent between the original study and replication study. Therefore we performed subsequent multivariate analyses with combined data (Table 5 and Table 6), in attempt to strengthen the statistical power.

**Table 5:** Odds Ratios (95% CI) of Type 2 Diabetes Prevalence, by Quartile of Plasma HO-1 Levels.

	Quartile of Plasma HO-1 Levels				
Variable	1 (Lowest)	2	3	4 (Highest)	*P* Value for Trend
Plasma HO-1 Levels, ng/ml	<0.63	0.63−1.14	1.14−2.06	≥2.06	/
Type 2 Diabetes Cases/Controls, n/n	43/152	57/153	130/153	351/153	/
Crude OR (95% CI)	1	1.32 (0.84−2.08)	3.00 (1.99−4.53)	8.11 (5.50−11.96)	<0.001
Adjusted OR (95% CI), Model 1	1	1.35 (0.86−2.14)	3.00 (1.98−4.55)	8.34 (5.63−12.36)	<0.001
Adjusted OR (95% CI), Model 2	1	1.36 (0.86−2.16)	3.05 (2.01−4.63)	8.23 (5.55−12.21)	<0.001
Adjusted OR (95% CI), Model 3a	1	1.68 (0.65−4.30)	2.87 (1.18−6.99)	4.89 (2.14−11.17)	<0.001
Adjusted OR (95% CI), Model 3b	1	1.64 (0.80−3.38)	2.60 (1.32−5.10)	5.03 (2.67−9.46)	<0.001
Adjusted OR (95% CI), Model 4	1	1.63 (0.62−4.28)	3.03 (1.23−7.50)	5.33 (2.29−12.43)	<0.001
Adjusted OR (95% CI), Model 5	1	1.61 (0.61−4.20)	2.99 (1.21−7.36)	5.35 (2.31−12.41)	<0.001
Adjusted OR (95% CI), Model 6	1	1.11 (0.35−3.49)	3.19 (1.11−9.14)	3.99 (1.48−10.75)	0.004

Results from multivariate Logistic regression analysis are presented using the combined data from the two-phase independent study.

Model 1, adjusted for age and sex.

Model 2, adjusted for Model 1, BMI and family history of diabetes;

Model 3a, adjusted for Model 2, FPG and FPI;

Model 3b, adjusted for Model 2, HOMA-beta and HOMA-IR.

Model 4, adjusted for Model 2, FPG, FPI, HOMA-beta and HOMA-IR.

Model 5, adjusted for Model 4, TC and TG.

Model 6, adjusted for Model 5, smoking, alcohol drinking, hypertension.

**Table 6:** Odds Ratios (95% CI) of Type 2 Diabetes Prevalence in Subgroups, by Quartile of Plasma HO-1 Levels.

	Quartile of Plasma HO-1 Levels					
Subgroups	1 (Lowest)	2	3	4 (Highest)	P Value for Trend	P Value for Interaction
**Age**						
<55 y	1	1.34 (0.76−2.34)	3.32 (1.99−5.53)	7.88 (4.87−12.75)	<0.001	0.745
≥55 y	1	1.42 (0.64−3.16)	2.64 (1.27−5.51)	9.17 (4.57−18.39)	<0.001	
**Sex**						
Female	1	2.37 (1.15−4.87)	3.12 (1.59−6.12)	8.43 (4.48−15.87)	<0.001	0.021
Male	1	0.88 (0.48−1.63)	3.02 (1.76−5.19)	8.41 (5.02−14.08)	<0.001	
**Overweight**						
No	1	1.14 (0.53−2.43)	2.63 (1.32−5.23)	8.23 (4.20−16.13)	<0.001	0.447
Yes	1	1.33 (0.72−2.44)	2.59 (1.47−4.56)	7.67 (4.55−12.93)	<0.001	
**Smoking**						
No	1	1.32 (0.69−2.53)	2.27 (1.24−4.16)	6.00 (3.45−10.44)	<0.001	0.243
Yes	1	1.15 (0.54−2.47)	3.55 (1.80−6.97)	9.36 (4.84−18.11)	<0.001	
**Drinker**						
No	1	1.12 (0.52−2.40)	2.58 (1.33−5.02)	8.46 (4.50−15.90)	<0.001	0.424
Yes	1	1.08 (0.54−2.16)	3.40 (1.78−6.45)	5.71 (3.14−10.37)	<0.001	
**Hypertension**						
No	1	1.04 (0.54−2.01)	2.64 (1.46−4.79)	10.99 (6.30−19.19)	<0.001	0.006
Yes	1	1.93 (0.85−4.39)	3.70 (1.76−7.77)	5.35 (2.63−10.87)	<0.001	

Results from multivariate Logistic regression analysis are presented using the combined data from the two-phase independent study.

Adjusted for age, sex, BMI and family history of diabetes.

Table 5 shows logistic analysis results of odds ratios (ORs) for New-T2DM associated with the level of plasma HO-1 concentrations, categorized into the quartiles according to its distribution in the controls. We observed increased ORs for New-T2DM associated with higher level of the plasma HO-1 concentration – likely a dose-response trend of this association. Participants in the highest quartile of plasma HO-1 concentrations, compared with the lowest, had a significantly increased ORs for New-T2DM (crude OR 8.11, 95% CI 5.50–11.96; *P* for trend <0.001). Adjustment for age and sex (adjusted OR 8.34, 95% CI 5.63–12.36; *P* for trend <0.001; Model 1) or further adjustment for BMI and family history of diabetes (adjusted OR 8.23, 95% CI 5.55–12.21; *P* for trend <0.001; Model 2) did not alter the results. Additional adjustment for FPG and FPI only moderately attenuated the association (adjusted OR 4.89, 95% CI 2.14–11.17, *P* for trend <0.001). The OR was 5.33 (95% CI 2.29–12.43, *P* for trend <0.001) after additional adjustment for HOMA-IR and HOMA-beta, 5.35 (95% CI 2.31–12.41, *P* for trend <0.001) after additional adjustment for TC and TG and 3.99 (95% CI 1.48–10.75, *P* for trend  = 0.004) after additional adjustment for smoking, alcohol drinking and hypertension.

Table 6 shows the results of analyses stratified by age, sex, overweight, smoking, alcohol drinking and hypertension for the association between HO-1 and New-T2DM. After adjustment for age, sex, BMI and family history of diabetes, high ORs for New-T2DM and dose-response relationship between plasma HO-1 concentration and increasing ORs of New-T2DM are also observed in all subgroups. Interaction effects were found between plasma HO-1 concentration and gender difference (*P* for interaction  = 0.021) as well as between plasma HO-1 concentration and hypertension (*P* for interaction  = 0.006); however, the reason for such interactions remains to be elucidated.

## Discussion

The interesting phenomenon that HO-1, rather than classical antioxidant enzymes, expression is significantly elevated in high glucose exposed pancreatic islet cells has attracted a panel of consequent studies including *in vitro*, *in vivo* and human studies. However, the current study, to our knowledge, is the first direct attempt to examine the relationship between circulating HO-1 concentrations and T2DM. Our study indicated that elevated plasma HO-1 concentrations were associated with an increased OR for New-T2DM, with strong dose-response trend. The association between plasma HO-1 concentrations and New-T2DM could not be explained by possible confounding effects from adjusting factors including age, sex, BMI, family history of diabetes, FPG, FPI, HOMA-beta and HOMA-IR, and it did not differentiates by age, sex and BMI. Therefore, these observations hold on in the analyses with the data of the first original study and the later replication study separately, making the observed association between plasma HO-1 concentrations and New-T2DM more convincing.

As an inducible stress protein, HO-1 is widely accepted to be a highly sensitive and reliable marker of oxidative stress [8], [26]. Reduced defense capacity against oxidative stress was found in cultured HO-1-deficient embryonic fibroblasts *in vitro*, HO-1-deficient mice *in vivo* [27], [28], and it was further confirmed in a typical human case with a complete loss of exon-2 of the maternal allele and a two-nucleotide deletion within exon3 of the paternal allele in HO-1 gene [29]. In contrast, up-regulation of HO-1 protein may represent an attempt to minimize cellular injury [30]. The current study demonstrated that plasma HO-1 concentrations were significantly increased in individuals with New-T2DM compared to controls. This is in parallel with our previous study which found predominant oxidative stress in the form of lipid peroxidation and DNA oxidative damage in New-T2DM [23]. Similar elevated HO-1 concentrations have been recently reported in other chronic disorders, such as chronic silicosis [14], Parkinson's disease [15] and hemophagocytic syndrome [16].

The sources and mechanisms of circulating HO-1 remain to be elucidated. Schipper et al postulated that plasma HO-1 may represent “leakage” of the enzyme from tissues to the plasma compartment analogous to the presence of circulating liver enzymes [31]. It was supported by subsequent investigations suggesting that elevated circulating HO-1 levels may derive from lesions of specific tissues [14], [32]. Because HO-1 is highly expressed in pancreatic islets or pancreatic beta cells [10]–[12], we therefore suppose that increased levels of HO-1 in plasma may result from “leakage” of injured pancreatic islets in individuals with New-T2DM. Obviously, confirmation for this hypothesis might be difficult in human studies; however, it could be carried out in a type 2 diabetic animal model.

Intracellular HO-1 gene expression in patients with T2DM seems to be complex since several relevant studies have yielded inconsistent results. It was reported that HO-1 gene expression was significantly increased in circulating monocytes [18] and lymphocytes [19] while decreased in muscular samples [20] and total leucocytes [21] in patients with T2DM. The reason for the inconsistent HO-1 levels in various cells or tissues remains unknown. HO-1 gene polymorphism among those studied population might contribute in some degree [17]. Another possible explanation might be HO-1 expression variation at different stages of diabetes, because HO-1 has been found to be increased in early stage of diabetes while decreased in late stage of diabetes [13]. However, it remains to be confirmed in longitudinal prospective investigations. It is also noteworthy that the current study found an elevation in plasma HO-1 concentration whereas our previous case-control study in which intracellular HO-1 protein expression in peripheral blood mononuclear cells was detected by flow cytometry in 606 healthy controls, 65 patients with impaired glucose regulation, and 217 patients with New-T2DM showed decreased HO-1 expression in peripheral blood mononuclear cells in New-T2DM [17]. This may be due to the possibility that plasma HO-1 concentrations in New-T2DM originated from other sources than peripheral blood mononuclear cells, which is similar with previous report [31].

Although our study showed strong association between plasma HO-1 and New-T2DM, it has several limitations. First, case-control study design does not allow us to establish a temporal relationship, so these findings should be confirmed in further prospective cohort studies. In addition, all participants in this study were of Chinese Han ethnicity, which minimizes the confounding effects by ethnic background. Whether these results can be generalized to other populations need to be studied further.

In conclusion, this study demonstrates a strong association between plasma HO-1 concentrations and ORs for New-T2DM – an apparent dose-response relationship that is independent of known risk factors for T2DM. Such findings add more knowledge regarding the important role of oxidative stress in T2DM. More consequent studies were warranted to confirm the clinical utility of plasma HO-1, especially in diagnosis and prognosis of T2DM and its complications.
